# Prevalence and associated factors of cognitive impairment among hospitalized older adults with depression: a cross-sectional study from a tertiary psychiatric hospital in Guangzhou, China

**DOI:** 10.3389/fpubh.2026.1869272

**Published:** 2026-07-14

**Authors:** Junrong Ye, Yuhan Zhang, Na Ma, Shengwei Wu, Jinrong Li, Jianxiong Guo, Aixiang Xiao

**Affiliations:** 1 The Affiliated Brain Hospital, Guangzhou Medical University, Guangzhou, China; 2 Key Laboratory of Neurogenetics and Channelopathies of Guangdong Province and the Ministry of Education of China, Guangzhou Medical University, Guangzhou, China; 3 School of Nursing, Guangzhou Medical University, Guangzhou, China

**Keywords:** cognitive impairment, cross-sectional study, depression, inpatient, older patients

## Abstract

**Background:**

Geriatric depression is frequently complicated by cognitive impairment, which is linked to impaired self-care ability, elevated dementia and suicide risks, and heavier disease burden. Its pathogenesis involves metabolic disorders, vascular injury, neurodegenerative changes, and clinical factors. Nutritional status, social support, and lifestyle remain unclear in older patients with depression. This study aims to determine the prevalence of cognitive impairment and identify factors associated with cognitive impairment in hospitalized older adults with depression.

**Methods:**

A cross-sectional study was conducted at a tertiary psychiatric hospital in China from February 2021 to March 2025. A total of 196 older patients diagnosed with depression were recruited using consecutive sampling. Cognitive impairment was evaluated using the Mini Mental State Examination (MMSE). Data collection included a general information questionnaire, Mini Nutritional Assessment Short Form (MNA-SF), Brief Psychiatric Rating Scale (BPRS), Geriatric Depression Scale (GDS), Social Support Rating Scale (SSRS), and Activities of Daily Living (ADL). Multivariable logistic regression was used to identify factors associated with cognitive impairment.

**Results:**

The sample was composed of 70.9% female participants, with a mean age of 69.24 ± 5.84 years. A total of 45.4% participants had cognitive impairment. The multivariable logistic regression analysis showed that better nutritional status (MNA-SF: *OR* = 0.223, *p* = 0.010) and higher ADL score (*OR* = 0.975, *p* = 0.021) were negatively associated with cognitive impairment, while higher BPRS score (*OR* = 1.049, *p* = 0.020) was positively associated with cognitive impairment.

**Conclusion:**

Cognitive impairment is prevalent in hospitalized older adults with depression. Poor nutrition, low self-care ability, and severity psychiatric symptoms are associated with a higher likelihood of cognitive impairment. Targeted intervention strategies should be developed to address these factors.

## 1 Introduction

As the global population ages rapidly, the disease burden of geriatric depression continues to rise (1). The prevalence of depression among adults aged 60 years and older worldwide was approximately 5.7% (2), while the prevalence among Chinese older adults reached 20.6% in 2023 (3). Geriatric depression not only involves core symptoms (down mood, loss of interest, sleep disturbance) but also impairs quality of life and self-care ability, while increases physical comorbidity risk, and social costs (4, 5). Beyond emotional and physical symptoms, cognitive impairment is common in older adults with depression. Epidemiological evidence showed that 20–50% of depressed patients present with cognitive impairment, including memory deficits, executive dysfunction, and impaired attention (6). Such impairment might interfere with instrumental and basic daily activities, including shopping, medication management, and self-care (7). Depressive symptoms in individuals with mild cognitive impairment have also been associated with a higher risk of later dementia (RR = 1.69) (8). Moreover, cognitive dysfunction combined with depressive symptoms would increase suicide risk (OR = 3.485) (9). These adverse outcomes complicate clinical management and increase disease-related burdens for patients.

The pathogenesis of cognitive impairment is multifactorial, with established risk factors grouped into three categories. In relation to vascular and metabolic factors, abnormal blood glucose, atrial fibrillation, overweight, and slow gait, were associated with incident cognitive impairment through metabolic dysregulation and vascular injury (10). In terms of comorbidities, Karam et al. (47) mentioned that chronic diseases such as hypertension and coronary heart disease could further aggravate cognitive impairment by inducing cerebrovascular disease and interfering with neurotransmitter metabolism (11). As for biological factors, Alafuzoff and Libard (12) found that diverse mixed neuropathological changes would be increased with age, with their cohort data showing dementia prevalence rose from 5% at 60–69 years to over 16% in individuals aged ≥80 years. Gender differences had also been reported, possibly reflecting hormonal, biological, and social factors (13). Finally, depression-related characteristics might play a role in cognitive impairment, as longer illness duration has been linked to structural and functional changes in cognitive-related networks, including prefrontal-hippocampal circuits (14, 15).

In addition to the above multifactorial risks, empirical findings suggested that modifiable behavioral and environmental factors played a pivotal role in cognitive health among older adults. Nutritional deficiencies, such as low levels of vitamin B12, folate, or omega-3 fatty acids, would impair neurotransmitter synthesis and exacerbate neuroinflammation, thereby accelerating cognitive decline (16, 17). Inadequate social support, often accompanied by loneliness, would dysregulate the hypothalamic pituitary adrenal axis and elevate cortisol levels, which was linked to reduced hippocampal volume and executive dysfunction (18, 19). Unhealthy lifestyle behaviors, including drinking and smoking, had been found to be associated with lower brain derived neurotrophic factor (BDNF) expression, reduced cerebral blood flow, and impaired amyloid clearance (20, 21). For hospitalized older adults with depression, these factors were particularly salient because depressive symptoms often led to poor appetite, social withdrawal, and disrupted daily routines, creating a vicious cycle that further compromised cognitive function (22–24). Thus, examining nutritional, social, and lifestyle variables in this understudied inpatient population would be important to identify actionable targets for clinical intervention.

Empirical studies mainly focused on community-dwelling older adults or the general older population, whereas hospitalized older adults with depression had received less attention. Hospitalization would be clinically important because patients admitted to psychiatric hospitals often have more severe symptoms, poorer functioning, treatment changes, and greater need for coordinated care. Moreover, as outlined above, nutritional status, social support, and lifestyle factors were mechanistically linked to cognitive health, these factors would be particularly compromised in hospitalized depressed patients due to depression-related appetite loss, social withdrawal, and disrupted routines. Limited study had systematically examined the associations of these modifiable factors with cognitive impairment specifically in hospitalized older adults with depression. Therefore, this study aimed to investigate the prevalence of cognitive impairment among hospitalized older adults with depression and to identify its demographic, clinical, nutritional, functional, social support, and lifestyle factors.

## 2 Methods

### 2.1 Subjects

This was a single-center, cross-sectional study. A total of 196 older patients with depression were consecutively recruited from geriatric inpatient wards of the Affiliated Brain Hospital of Guangzhou Medical University during the period from February 2021 to March 2025. Inclusion criteria: (1) age ≥60 years old; (2) depression was diagnosed by qualified psychiatrists according to the International Classification of Diseases, Tenth Revision (ICD-10), based on clinical interviews and medical records; (3) being able to understand and complete all questionnaires and assessments independently without assistance; (4) provide written informed consent. Exclusion criteria: (1) dementia or other primary neurodegenerative disorders documented in medical records or identified by clinical assessment; (2) being diagnosed with critical hearing impairment that was unable to communicate verbally during face-to-face interviews; (3) severe, unstable, acute, or terminal non-communicable diseases, including cardiovascular, hepatic, or renal diseases, judged by the treating clinicians to interfere with study participation or assessment completion.

### 2.2 Questionnaires

The instruments were selected to cover the major domains relevant to cognitive impairment in hospitalized older adults with depression, including cognition (MMSE), nutritional status (MNA-SF), broad psychiatric symptom severity (BPRS), depressive symptoms specific to older adults (GDS), perceived and objective social support (SSRS), and functional independence in daily activities (ADL).

#### 2.2.1 General information questionnaire

A general information questionnaire was used to collect demographic and clinical data, including age, gender, education, marital status, drinking behavior, smoking behavior, duration of depression, first admission, comorbidity, number of current antipsychotic medications, primary caregiver (defined as the individual who provided the most daily care; caregiver status was categorized into three groups: self-care, professional caregiver, and family caregiver), and hospital stay duration.

#### 2.2.2 Mini mental state examination (MMSE)

The MMSE, developed by Folstein et al. (25), was used to assess cognitive impairment. The scale has a maximum total score of 30 points, and a higher score signifies superior cognitive function. Education-adjusted cutoff scores were used to define screen-positive cognitive impairment: MMSE ≤24 for participants with secondary education or above, ≤20 for participants with primary education, and ≤17 for illiterate participants.

#### 2.2.3 The short-form mini nutritional assessment (MNA-SF)

The MNA-SF developed by Rubenstein et al. in 2001, was utilized to assess nutritional status (26). The scale comprises six items with a total score of 0–14. Malnutrition was defined as a score <12, and normal nutrition as ≥12.

#### 2.2.4 Brief psychiatric rating scale (BPRS)

The Brief Psychiatric Rating Scale was utilized to evaluate psychiatric symptoms (27). BPRS covers a broad range of psychiatric manifestations, including somatic concern, anxiety, depressive mood, hostility, guilt, tension, grandiosity, suspiciousness, hallucinatory behavior, unusual thought content, bizarre behavior, withdrawal, retardation, motor hyperactivity, uncooperativeness, blunted affect, excitement, and disorientation. The total score ranges from 18 to 126 points, with higher scores indicating more severe psychiatric symptoms.

#### 2.2.5 Geriatric depression scale (GDS)

The GDS introduced by Brink and Yesavage et al. (28) was designed specifically for evaluating depressive symptoms in older adults. The 30-item scale includes items such as depressed mood, reduced activity, agitation, and self-reported pain, among other indicators. The scoring criteria are: 0–10 points indicated no or minimal depressive symptoms, 11–20 points indicated mild depressive symptoms, and 21–30 points indicated moderate to severe depressive symptoms (29).

Although both GDS and BPRS assess psychiatric symptoms, they capture different constructs. The GDS focuses on depressive symptoms in older adults, whereas the BPRS provides a broader assessment of psychiatric symptom severity, including anxiety, thought disturbance, suspiciousness, disorientation, and other symptoms that may be relevant to cognition.

#### 2.2.6 Social support rating scale (SSRS)

The SSRS was employed to evaluate the level of the social support from three dimensions, including objective support (3 items), subjective support (4 items), and the utilization of social support (3 items) (30). The total score ranges from 10 to 40, with higher scores reflecting greater level of social support.

#### 2.2.7 Activities of daily living scale (ADL)

The ADL scale, created by Mahoney and Barthel in 1965, was used to assess daily functioning (31). This 10-item tool defines four functional levels based on both the patient’s need for help and its severity. A higher score signifies that the patient is more self-reliant and less dependent.

### 2.3 Data collection

This study was conducted in a tertiary-level public psychiatric hospital. Prior to data collection, all interviewers underwent a standardized training program on the study purpose, demonstration interviews, scale administration, scoring criteria, and supervised practice assessments. Inter-rater reliability was tested using intraclass correlation coefficients (ICC) for all main scales. The ICC values were 0.91 for MMSE, 0.89 for MNA-SF, 0.92 for BPRS, 0.90 for GDS, 0.88 for SSRS, and 0.93 for ADL, indicating good consistency (32). Researchers explained the study objectives, procedures, and confidentiality protections before obtaining written informed consent from participants. All assessments were performed via interviews in a quiet and private room. Demographic and clinical variables, including age, education, marital status, admission status, medication type, comorbidity status, smoking and drinking behavior, and length of stay, were extracted from medical records. The average duration of each assessment was approximately 30 to 40 min. For incomplete or unclear items, investigators re-explained the questions on site and checked responses immediately. No missing data were present in final analysis dataset. Then interviews with participants were conducted during the first 3 days of admission to minimize the influence of acute treatment interventions, drug adjustment, and clinical status changes on cognitive function, psychiatric symptoms, and scale scores (33).

### 2.4 Statistical analysis

Data analysis was performed using SPSS statistical software version 29.0. Measurement data were expressed as mean ± standard deviation, and comparisons were made using the t-test. Count data were presented as frequency and percentage [*n* (%)], with comparisons conducted via the *χ*^2^ test. No missing data were observed in final analysis dataset after quality control and verification.

The dependent variable in the logistic regression model was cognitive impairment, which was coded as 1 = presence of cognitive impairment and 0 = absence of cognitive impairment based on education adjusted MMSE cutoff scores. Logistic regression analysis was applied to explore the influencing factors of cognitive impairment in late-life depression. Variable selection for multivariable analysis followed a two stage approach. First, candidate variables were identified using univariable logistic regression with a threshold of *p* < 0.05. Second, to minimize residual confounding, clinically relevant variables (age, sex, education level, course of depression, comorbidity, and type of antidepressant medication) were forced into the multivariable model regardless of their univariate *p*-value. The final model was fitted using the enter method (34).

The Box-Tidwell test was used to verify the linearity assumption between continuous variables and the logit of cognitive impairment in the logistic regression model (35). All interaction terms were non-significant (*p* > 0.05), indicating that the linearity assumption was met for all continuous predictors. Multicollinearity was assessed using the variance inflation factor (VIF). VIF > 5 was considered suggestive of problematic multicollinearity and VIF > 10 was considered severe multicollinearity (36). Model fit was evaluated using the Hosmer-Lemeshow goodness of fit test (*p* > 0.05), and the overall significance of the model was assessed with the Omnibus test (37).

## 3 Results

### 3.1 Baseline demographic characteristics and clinical outcomes

Among the 196 hospitalized older adults with depression, 89 screened positive for cognitive impairment, corresponding to a prevalence of 45.4%. The mean age was 69.24 ± 5.84 years, 70.9% were female, 65.8% had secondary education or above, 7.1% had a history of smoking, 6.6% had a history of drinking, and 48.0% used two or more antidepressants. Table 1 showed the demographic features and selected clinical results of patients with cognitive impairment compared to those with normal cognition. No statistically significant differences were found in terms of gender, age, educational background, marital status, comorbidity or primary caregivers (*P >* 0.05). In comparison to the normal group, patients in the cognitive impairment group exhibited a higher rate of drinking behavior (*p* = 0.018) and more frequent occurrences of malnutrition (*p* < 0.001). Regarding psychiatric symptoms, the cognitive impairment group showed significantly greater psychiatric severity as measured by the BPRS (*p* < 0.001). Additionally, the normal group demonstrated better social support (SSRS) (*p* = 0.002) and superior abilities in performing daily activities (ADL) (*p* < 0.001).

**Table 1:** Baseline demographic and clinical characteristics of depressed older patients with and without cognitive impairment.

Characteristics	Total (*n* = 196)	Impaired (*n* = 89)	Normal (*n* = 107)	Statistic	Test level
	Mean (SD)			*t*	*p*
Age	69.24(5.84)	69.73(6.35)	68.83(5.37)	−1.075	*p =* 0.284
Course of depression	8.81(10.33)	8.82(10.44)	8.81(10.30)	−0.012	*p =* 0.991
Brief Psychiatric Rating Scale (BPRS)	33.58(9.58)	36.51(10.33)	31.14(8.19)	−4.054	***P* < 0.001**
Geriatric Depression Scale (GDS)	18.93(7.74)	19.80(7.39)	17.89(8.05)	−1.729	*p =* 0.085
Social Support Rating Scale (SSRS)	34.11(6.88)	32.41(5.90)	35.50(7.34)	3.198	***P =* 0.002**
Activities of Daily Living (ADL)	86.56(18.25)	80.73(22.10)	91.40(12.45)	4.250	***P* < 0.001**

Characteristics		n (%)			*χ^2^*	*p*
Sex	Male	57 (29.1%)	28 (31.5%)	29 (27.1%)	(Reference)	
	Female	139 (70.9%)	61 (68.5%)	78 (72.9%)	0.447	*p =* 0.504
Comorbidity	No	20 (10.2%)	9 (10.1%)	11 (10.3%)	(Reference)	
	Yes	176 (89.8%)	80 (89.9%)	96 (89.7%)	0.001	*p =* 0.969
Education	Primary or below	67 (34.2%)	28 (31.5%)	39 (36.4%)	(Reference)	
	Secondary or higher	129 (65.8%)	61 (68.5%)	68 (63.6%)	0.537	*p =* 0.464
Marital status^1^	Single	42 (21.4%)	18 (20.2%)	24 (22.4%)	(Reference)	
	Couple	154 (78.6%)	71 (79.8%)	83 (77.6%)	0.140	*p =* 0.708
Primary caregiver	Self-care	38 (19.4%)	19 (21.3%)	19 (17.8%)	(Reference)	
	Professional caregiver	14 (7.1%)	7 (7.9%)	7 (6.5%)		
	Family Caregivers	144 (73.5%)	63 (70.8%)	81 (75.7%)	0.602	*p =* 0.740
First admission	No	127 (64.8%)	57 (64.0%)	70 (65.4%)	(Reference)	
	Yes	69 (35.2%)	32 (36.0%)	37 (34.6%)	0.040	*p =* 0.841
Smoking	No	182 (92.9%)	82 (92.1%)	100 (93.5%)	(Reference)	
	Yes	14 (7.1%)	7 (7.9%)	7 (6.5%)	0.128	*p =* 0.720
Drinking	No	183 (93.4%)	79 (88.8%)	104 (97.2%)	(Reference)	
	Yes	13 (6.6%)	10 (11.2%)	3 (2.8%)	5.579	***P =* 0.018**
Length of stay	≤ 28 days	145 (74.0%)	65 (73.0%)	80 (74.8%)	(Reference)	
	>28 days	51 (26.0%)	24 (27.0%)	27 (25.2%)	0.076	*p =* 0.783
Types of antidepressants	0 type	17 (8.7%)	8 (9.0%)	9 (8.4%)	(Reference)	
	1 type	85 (43.3%)	37 (41.6%)	48 (44.9%)		
	≥2 type	94 (48.0%)	44 (49.4%)	50 (46.7%)	0.784	*p =* 0.676
Mini Nutritional Assessment Short-Form (MNA-SF)	Malnutrition	165 (84.2%)	85 (95.5%)	80 (74.8%)	(Reference)	
	Normal nutrition	31 (15.8%)	4 (4.5%)	27 (25.2%)	15.695	***P* < 0.001**

^1^The category “couple” includes both legally married individuals and people living with a partner. Bold values represent statistically significant results with *p* < 0.05.

### 3.2 Variables associated with cognitive impairment in older patients with depression

Univariable logistic regression analysis was conducted with screen positive cognitive impairment as the dependent variable to explore potential associated factors (Table 2). The results demonstrated that higher BPRS score (*OR* = 1.069, *95% CI:* 1.032–1.107, *p* < 0.05), lower SSRS score (*OR* = 0.932, *95% CI*: 0.891–0.975, *p* < 0.05), lower ADL score (*OR* = 0.963, *95% CI*: 0.945–0.982, *p* < 0.05), current drinking behavior (OR = 4.388, *95% CI*: 1.169–16.475, *p* < 0.05), and normal nutritional status (*OR* = 0.139, *95% CI*: 0.047–0.416, *p* < 0.05) were significantly associated with cognitive impairment. In contrast, age, course of depression, GDS score, sex, educational level, marital status, primary caregiver type, first admission status, smoking behavior, length of hospital stay, and type of antidepressant medication showed no significant associations with cognitive impairment in univariate analysis (*p* > 0.05).

**Table 2:** Univariable logistic regression analysis of factors associated with cognitive impairment in older adults with depression.

Variables		OR	*P*	95% CI
Age		1.027	*p =* 0.283	(0.978–1.078)
Course of depression		1.000	*p =* 0.990	(0.973–1.028)
BPRS		1.069	***P*<0.001**	(1.032–1.107)
GDS		0.968	*p =* 0.086	(0.933–1.005)
SSRS		0.932	***P =* 0.002**	(0.891–0.975)
ADL		0.963	***P*<0.001**	(0.945–0.982)
Sex	Male	(Reference)		
	Female	0.810	*P =* 0.504	(0.437–1.503)
Comorbidity	No	(Reference)		
	Yes	0.969	*p =* 1.019	(0.402–2.580)
Education	Primary or less	(Reference)		
	Secondary or higher	1.249	*P =* 0.464	(0.689–2.267)
Marital status	Single	(Reference)		
	Coupled	1.141	*P =* 0.708	(0.573–2.270)
Primary caregiver	Self-care	(Reference)		
	Professional caregiver	0.778	*p =* 0.654	(0.259–2.332)
	Family Caregivers	1.000	*p =* 1.000	(0.294–3.406)
First admission	No	(Reference)		
	Yes	1.062	*P =* 0.841	(0.590–1.913)
Drinking	No	(Reference)		
	Yes	4.388	***p =* 0.028**	(1.169–16.475)
Smoking	No	(Reference)		
	Yes	1.220	*p =* 0.721	(0.411–3.618)
Length of stay	≤28 days	(Reference)		
	>28 days	1.094	*P =* 0.783	(0.577–2.075)
Types of antidepressants	0 type	(Reference)		
	1type	0.653	*p =* 0.424	(0.230–1.857)
	≥2 type	0.782	*p =* 0.642	(0.278–2.202)
Nutrition (MNA-SF)	Malnutrition	(Reference)		
	Normal nutrition	0.139	***P*<0.001**	(0.047–0.416)

Bold values represent statistically significant results with *p* < 0.05.

### 3.3 Multivariable analysis of cognitive impairment in older patients with depression

The overall model was statistically significant (Omnibus *χ^2^* = 42.339, *df* = 11, *p* < 0.05), indicating that the combination of predictors was significantly associated with cognitive impairment. The Hosmer-Lemeshow goodness of fit test showed no significant difference between observed and predicted outcomes (*χ^2^* = 6.292, *df* = 8, *P* > 0.05), confirming adequate model fit. All *VIF* values ranged from 1.033 to 1.186, with no value exceeding 2, indicating no substantial multicollinearity among variables. The Box-Tidwell test confirmed that the linearity assumption between continuous predictors (BPRS, SSRS, ADL, age, course of depression) and the logit of cognitive impairment was satisfied (*p* > 0.05). Consequently, all continuous variables were entered in their original form into the final model.

Variables that showed significant associations in the univariable analysis (*p* < 0.05) were included in multivariable logistic regression model, with adjustment for clinically relevant covariates including age, sex, educational level, course of depression, comorbidity, and type of antidepressant medication. After sensitivity analysis, the drinking variable was excluded from the multivariable model due to sparse data, extremely wide confidence intervals, and potential unstable estimation caused by the small sample size (*n* = 13). The exclusion of this variable did not alter the direction and statistical significance of the main associated factors (MNA-SF, ADL, and BPRS) (Table 3).

**Table 3:** Multivariable logistic regression analysis of factors associated with cognitive impairment in older adults with depression.

Variables		*β*	Wald *χ*^2^	SE	Adjusted OR	*P*	95% CI
Age		0.021	0.471	0.031	1.022	*p =* 0.492	(0.961–1.086)
Course of depression		0.008	0.232	0.017	1.008	*p =* 0.630	(0.976–1.041)
ADL		−0.025	5.287	0.011	0.975	***P =* 0.021**	(0.955–0.996)
SSRS		−0.043	2.812	0.026	0.958	*p =* 0.094	(0.910–1.007)
BPRS		0.047	5.406	0.020	1.049	***P =* 0.020**	(1.007–1.091)
Sex	Male	(Reference)					
	Female	−0.146	0.157	0.364	0.864	*p =* 0.692	(0.419–1.782)
Nutrition (MNA-SF)	Malnutrition	(Reference)					
	Normal nutrition	−1.499	6.615	0.583	0.223	***P =* 0.010**	(0.071–0.700)
Education	Primary or less	(Reference)					
	Secondary or higher	0.474	1.697	0.364	1.607	*p =* 0.193	(0.787–3.281)
Types of antidepressants	0 type	(Reference)					
	1type	−0.221	0.123	0.632	0.801	*p =* 0.726	(0.232–2.764)
	≥2 type	0.078	0.016	0.621	1.081	*p =* 0.900	(0.320–3.654)
Comorbidity	No	(Reference)					
	Yes	−0.035	0.004	0.537	0.966	*p =* 0.948	(0.337–2.767)

Bold values represent statistically significant results with *p* < 0.05.

After adjustment, three factors remained independently associated with screen positive cognitive impairment: ADL, BPRS, and MNA-SF. Specifically, each one-point increase in ADL score was associated with a 2.5% decrease in the odds of cognitive impairment (*OR* = 0.975, *95% CI*: 0.955–0.996, *p* = 0.021), each one point increase in BPRS score was associated with a 4.9% increase in the odds of cognitive impairment (*OR* = 1.049, *95% CI*: 1.007–1.091, *p* = 0.020), and normal nutritional status assessed by MNA-SF, compared with malnutrition, was associated with significantly lower odds of cognitive impairment (*OR* = 0.223, *95% CI*: 0.071–0.700, *p* < 0.010). Additionally, age, sex, educational level, course of depression, comorbidity, and type of antidepressant medication had not been identified as independent associated factors in the final adjusted model (*p* > 0.05).

## 4 Discussion

This study demonstrated a high prevalence of cognitive impairment (45.4%) among hospitalized older patients with depression, meanwhile nutritional status, ADL score, and BPRS score were associated with the odds of cognitive impairment. This figure fell within the 20–50% range documented in prior studies but was higher than the rate reported by Li et al. (38) (45.4% vs. 36.4%) (6). This difference might be due to the study settings and participant characteristics, as the present study included only hospitalized patients with depression, whereas previous research included both inpatients and community dwelling individuals.

Normal nutritional status was associated with a lower likelihood of cognitive impairment in this study. This observation aligns with existing literature that linked greater nutritional status to more favorable cognitive outcomes in older adults (39). Potential underlying associations involved support for neurotransmitter synthesis, maintenance of cerebrovascular health, and regulation of the gut-brain axis (40–42). These processes might be linked to reduced central inflammatory activity and stable neural function, which in turn were associated with preserved cognitive performance (43). Therefore, routine nutritional screening and targeted nutritional support would be beneficial in clinical care for older patients with depression. Future researches are expected to investigate optimal nutritional components and long-term intervention effects to better support cognitive health in this vulnerable population.

Being consistent with the findings by Edwards et al. (22), a marked inverse relationship was observed between cognitive function and activities of daily living (ADL) in older adults. Specifically, individuals exhibiting a lower level of cognitive function demonstrated a greater likelihood of declined ADL. Given the cross-sectional design of this study, though temporal sequence cannot be determined, lower ADL capacity might represent a consequence of cognitive impairment rather than a contributing factor. Cognitive status had been linked to physical frailty in older adults, and reduced independence in daily activities might further restrict social engagement and environmental stimulation (44). These observations highlighted the complex and reciprocal associations between cognitive function and functional status in older patients with depression.

Noticeably, higher BPRS scores were associated with greater cognitive impairment risk in older depressed patients, whereas GDS scores showed no significant correlation. This difference reflected that high homogeneity of depressive symptoms in hospitalized participants cohort, whereas BPRS captured broader psychiatric symptoms including thought disturbance and inattention, which were linked to neurostructural changes of cognitive impairment, such as white matter lesions and hippocampal atrophy (45, 46). Clinically, close cognitive function monitoring and prioritized screening would be crucial for older depressed patients with prominent psychiatric symptoms.

Social support, measured by SSRS, was significantly associated with cognitive impairment in univariable analysis but showed no statistic difference after multivariable adjustment. This might due to the fact that the effect of social support was largely mediated or confounded by daily functioning, nutritional status, and psychiatric symptoms. Although an independent effect of social support on cognitive impairment was not observed in this study, previous studies reported that individuals with lower level of cognitive impairment exhibited higher level of social support (18), thus future studies should further adopt longitudinal designs to clarify the specific pathways and key mediating variables through which social support influences cognitive function.

Limitations of this study should be noted. Our conclusion should be interpreted with cautions because the cognitive status was assessed by MMSE, therefore the outcome reflected screen-positive cognitive impairment rather than clinical diagnosis. Some MNA-SF items were overlapped with cognitive, psychiatric, and functional status, thus future study would be needed to examine the independent association between nutrition and cognitive impairment. Besides, the cross-sectional design could not determine temporal or causal relationships, and the limitations of small sample size might influence the statistical power and sparsity bias, thus above factors might influence the generalizability of our conclusion in community dwelling or outpatient populations.

## 5 Conclusion

This study indicated that 45.4% of hospitalized older adults with depression screened positive for cognitive impairment. Cognitive impairment was associated with nutritional status, activities of daily living (ADL), and psychiatric symptom severity in this population. These findings provided evidence for early identification and targeted intervention of cognitive impairment in this population.

## Data Availability

The raw data supporting the conclusions of this article will be made available by the authors, without undue reservation.
